# Three new species of the genus *Lathrobium* Gravenhorst (Coleoptera, Staphylinidae, Paederinae) from the Jiulongshan Natural Reserve, East China

**DOI:** 10.3897/zookeys.184.2634

**Published:** 2012-04-21

**Authors:** Zhong Peng, Li-Zhen Li, Mei-Jun Zhao

**Affiliations:** 1 Department of Biology, College of Life and Environmental Sciences, Shanghai Normal University, Shanghai, 200234, P. R. China

**Keywords:** Coleoptera, Staphylinidae, taxonomy, *Lathrobium*, new species, key to species, Jiulongshan, Zhejiang, China

## Abstract

Three new species of the genus *Lathrobium* Gravenhorst from Jiulongshan Natural Reserve, Zhejiang, East China, *Lathrobium jiulongshanense* **sp. n.**, *Lathrobium sheni* **sp. n.** and *Lathrobium zhaotiexiongi* **sp. n.** are described and illustrated. A key to the *Lathrobium* species from Zhejiang Province is provided.

## Introduction

Up to today, eight species have been recorded from Zhejiang Province: *Lathrobium imadatei* Watanabe & Luo, 1992 and *Lathrobium tamurai* Watanabe & Luo, 1992 from Wuyanling Natural Reserve, *Lathrobium cooteri* Watanabe, 1999 from the Lin-long Shan Mountain, *Lathrobium rougemonti* Watanabe, 1999 and *Lathrobium tianmushanense* Watanabe, 1999 from the West Tianmushan Mountain, and *Lathrobium lingae* Peng, Li & Zhao, 2012, *Lathrobium longwangshanense* Peng, Li & Zhao, 2012 and *Lathrobium uncum* Peng, Li & Zhao, 2012 from the Longwangshan Mountain. Jiulongshan is a Natural Reserve located in southwestern Zhejiang, and no *Lathrobium* species have been reported from there.

In 2006, our colleagues made a collecting trip to the Jiulongshan Natural Reserve (at. 28°21'N, 118°53'E), and obtained a large number of *Lathrobium* specimens. Three species were recognized and all of them are new to science. In addition, some specimens collected recently in Zhuji City (approximately 29°43'N, 119°59'E) were provided by Mr. Tie-Xiong Zhao, all of them are belonging to one of the new species mentioned above.

A map (Fig. 5) showing the distribution of *Lathrobium* in Zhejiang and a key is provided.

## Material and methods

All specimens were collected from the leaf litter of the forest floor by sifting. The following abbreviations are used in the text, with all measurements in millimeters:

BLbody length: length of body from the labral anterior margin to the anal apex;


HLhead length: length of head from the clypeal anterior margin to the posterior margin of the head;


HWhead width: maximum width of head;


PLpronotum length: length of pronotum along midline;


PWpronotum width: maximum width of pronotum;


ELelytra length: length of elytra from the apex of the scutellum to the elytral posterior margin.


The type materials is deposited in the Insect Collection of Shanghai Normal University (SNUC).

## Descriptions

### Lathrobium jiulongshanense

Peng & Li sp. n.

urn:lsid:zoobank.org:act:558DE96D-6F1C-4A37-8BDE-C642DD835556

http://species-id.net/wiki/Lathrobium_jiulongshanense

Figs 1A 2

#### Type locality.

Jiulongshan Natural Reserve, Zhejiang Province, East China

#### Type material

(22 ♂♂, 20 ♀♀)**.** Holotype: ♂, labeled ‘**CHINA:** Zhejiang Prov. / Suichang County / Jiulongshan N. R. / 31.vii.2006, alt. 500–700 m / Li & Shen leg.’. Paratypes: 13 ♂♂, 7 ♀♀, same label data as holotype; 8 ♂♂, 12 ♀♀, same, except ‘30.v.2006’; 1♀, same, except ‘28.v.2006’.

#### Description.

Measurements and ratios:BL 8.90–10.08, HL 1.43–1.52, HW 1.56–1.61, PL 1.80–1.92, PW 1.55–1.71, EL 1.09–1.18, HW/HL 0.95–1.11, HW/PW 0.94–1.02, HL/PL 0.77–0.81, PL/PW 1.10–1.15, EL/PL 0.61–0.66.

**Figure 1.**
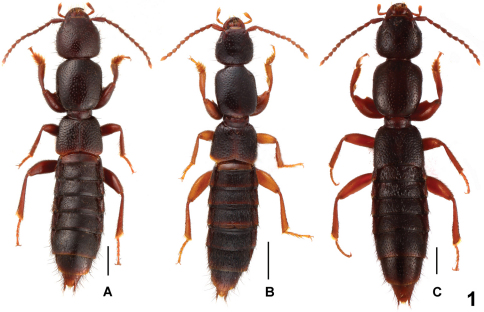
Male habitus of *Lathrobium* spp., **A** *Lathrobium jiulongshanense* **B** *Lathrobium sheni* **C***Lathrobium zhaotiexiongi*. Scales: 1.0 mm.

Habitus as in Fig. 1A. Body brown with paler apex, legs brown, antennae brown to reddish brown.

Head subquadrate; punctation coarse and moderately sparse; interstices with shallow microsculpture; eyes small, usually approximately 1/3–3/8 the length of postocular region in dorsal view.

Pronotum with lateral margins weakly convex in dorsal view; punctation sparser than that of head; impunctate midline narrow; interstices without microsculpture.

Elytra with punctation denser than that of pronotum and well-defined; hind wings reduced.

Abdomen with dense punctation; interstices with shallow microsculpture.

**Figure 2.**
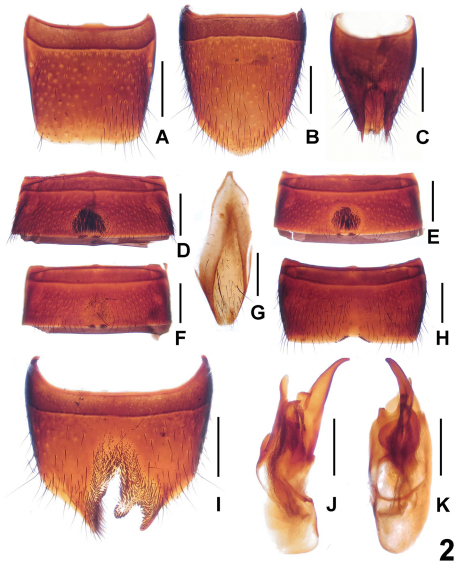
*Lathrobium jiulongshanense*. **A** female tergite VIII**B** female sternite VIII **C** female tergites IX–X**D** male sternite IV **E** male sternite V **F** male sternite VI **G** male sternite IX **H** male sternite VII **I** male sternite VIII **J** aedeagus in lateral view **K** aedeagus in ventral view. Scales: 0.5 mm.

Male. Sternite IV (Fig. 2D) and V (Fig. 2E) with dense short darkish setae in postero-median concavity, on posterior margin with 6–10 peg-like setae; sternite VI (Fig. 2F) similar to V, but with much sparser setae in median concavity; sternite VII (Fig. 2H) with narrow impression, on either side of this impression with 9–12 peg-like setae; sternite VIII (Fig. 2I) with different length of setae surrounding distinctly asymmetric and deep emargination in postero-median portion; sternite IX (Fig. 2G) asymmetric; aedeagus (Fig. 2J, 2K) with conspicuously long ventral process and short dorsal sclerite.

Female. Posterior margin of tergite VIII (Fig. 2A) truncate; sternite VIII (Fig. 2B) longer than that of male, posterior margin broadly convex and with micropubescence; tergite IX (Fig. 2C) not separated from X (Fig. 2C) with short lateral processes.

#### Distribution.

East China (Zhejiang: Jiulongshan Natural Reserve).

#### Etymology.

The species is named after its type locality.

#### Remarks.

The new species is distinguished from all its congeners in the following points: male sternites IV–VI with several peg-like setae at the posterior margin; male sternite VIII with the different length of setae surrounding the asymmetric and relatively deep emargination.

### Lathrobium sheni

Peng & Li sp. n.

urn:lsid:zoobank.org:act:33725E9C-7290-48A9-9194-20B31850D88A

http://species-id.net/wiki/Lathrobium_sheni

Figs 1B 3

#### Type locality.

Jiulongshan Natural Reserve, Zhejiang Province, East China

#### Type material

(2 ♂♂, 1 ♀)**.** Holotype: ♂, labeled ‘**CHINA:** Zhejiang Prov. / Suichang County / Jiulongshan N. R. / 31.vii.2006, alt. 500–700 m / Li & Shen leg.’. Paratypes: 1 ♂, 1 ♀, same label data as holotype.

#### Description.

Measurements and ratios:BL 6.12–7.51, HL 0.83–0.93, HW 0.93–1.02, PL 1.15–1.26, PW 1.00–1.11, EL 0.74–0.83, HW/HL 0.93–1.12, HW/PW 0.90–0.93, HL/PL 0.72–0.81, PL/PW 1.14–1.17, EL/PL 0.64–0.66.

Habitus as in Fig. 1B. General appearance similar to *Lathrobium jiulongshanense*, except for the lighter coloration of the legs, the much smaller body size, and the sparser punctation on head and pronotum.

**Figure 3.**
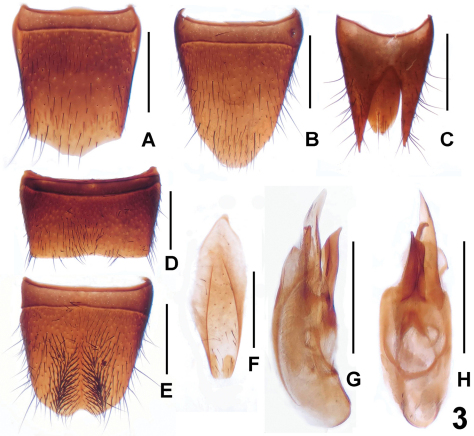
*Lathrobium sheni*. **A** female tergite VIII**B** female sternite VIII **C** female tergites IX–X**D** male sternite VII **E** male sternite VIII **F** male sternite IX **G** aedeagus in lateral view **H** aedeagus in ventral view. Scales: 0.5 mm.

Male. Posterior margin of sternite VII (Fig. 3D) weakly concave; sternite VIII (Fig. 3E) with two rows of dense setae; sternite IX (Fig. 3F) almost symmetric; aedeagus (Fig. 3G, 3H) with short ventral process and longer dorsal sclerites.

Female. Posterior margin of tergite VIII (Fig. 3A) pointed in middle; sternite VIII (Fig. 3B) much longer than that of male, posterior margin strongly convex; tergite IX (Fig. 3C) narrowly separated from X (Fig. 3C) and with slender lateral processes.

#### Distribution.

East China (Zhejiang: Jiulongshan Natural Reserve).

#### Etymology.

The species is named after Shan-Jia Shen, collector of the type specimens.

#### Remarks.

This species resembles *Lathrobium tamurai* Watanabe, 1992, which too was described from Zhejiang Province, in having an aedeagus with a short and apically hook-shaped ventral process. It resembles *Lathrobium yinae* Watanabe, 1997 from Yunnan in having the posterior margin of the male sternite VII weakly concave and two rows of dense setae on male sternite VIII. The new species can be readily distinguished from these species by two rows of dense setae on the male sternite VIII and by the much shorter ventral process of the aedeagus. In *Lathrobium tamurai*, the male sternite VIII has short darkish setae. In *Lathrobium yinae*, the ventral process of the aedeagus is elongate.

### Lathrobium zhaotiexiongi

Peng & Li sp. n.

urn:lsid:zoobank.org:act:F4B95489-78F5-4D69-95E5-B2BEB7342939

http://species-id.net/wiki/Lathrobium_zhaotiexiongi

Figs 1C 4

#### Type locality.

Jiulongshan Natural Reserve, Zhejiang Province, East China

#### Type material

(7 ♂♂, 5 ♀♀)**.** Holotype: ♂, labeled ‘**CHINA:** Zhejiang Prov. / Suichang County / Jiulongshan N. R. / 28.vii.2006, alt. 500–600 m / Li & Shen leg.’. Paratypes: 2 ♂♂, 3 ♀♀, same label data as holotype; 4 ♂♂, 2 ♀♀, same data, except ‘Zhuji City / Majian Town / 16.x.2011, alt. 200 m / Tie-Xiong Zhao leg.’.

#### Description.

Measurements and ratios:BL 9.90–11.12, HL 1.36–1.43, HW 1.49–1.58, PL 1.74–1.80, PW 1.56–1.61, EL 1.05–1.14, HW/HL 1.07–1.14, HW/PW 0.94–0.98, HL/PL 0.76–0.80, PL/PW 1.12–1.13, EL/PL 0.59–0.65.

Habitus as in Fig. 1C. Generally similar to *Lathrobium jiulongshanense*, except for the lighter coloration of the legs, the somewhat larger body size, and the moderately sparse punctation on head and pronotum.

**Figure 4.**
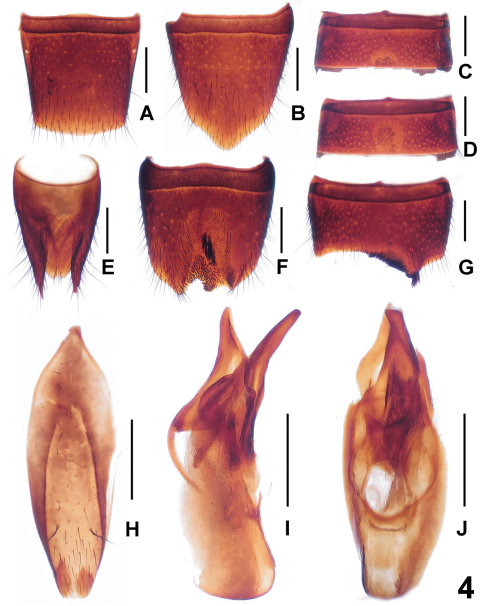
*Lathrobium zhaotiexiongi*. **A** female tergite VIII**B** female sternite VIII **C** male sternite V**D** male sternite VI **E** female tergites IX–X **F** male sternite VIII **G** male sternite VII **H** male sternite IX **I** aedeagus in lateral view **J** aedeagus in ventral view. Scales: 0.5 mm.

Male. Sternite V (Fig. 4C) with dense short darkish setae in posterior concavity; sternite VI (Fig. 4D) similar to sternite V, but with slightly shorter setae in median concavity; sternite VII (Fig. 4G) with different length of setae surrounding the distinctly asymmetric emargination in posterior portion; sternite VIII (Fig. 4F) with different length of setae in large impression and shallow posterior emargination; sternite IX (Fig. 4H) almost asymmetric; aedeagus (Fig. 4I, 4J) with short ventral process and dorsal sclerite.

Female. Posterior margin of tergite VIII (Fig. 4A) slightly convex; sternite VIII (Fig. 4B) longer than that of male, posterior margin distinctly pointed in the middle; tergite IX (Fig. 4C) narrowly separated from X (Fig. 4C) and with moderately slender lateral processes.

#### Distribution.

East China (Zhejiang: Jiulongshan Natural Reserve and Majian Town).

#### Etymology.

The species is named after Tie-Xiong Zhao, collector of the type series, a 12-year-old boy who is most enthusiastic about collecting beetles.

#### Remarks.

From other *Lathrobium* species of the Jiulongshan N. R., *Lathrobium zhaotiexiongi* is readily separated by the male sternite VII having setae of variable length surrounding the posterior emargination and the male sternite VIII having setae of variable length in the postero-median impression.

**Figure 5.**
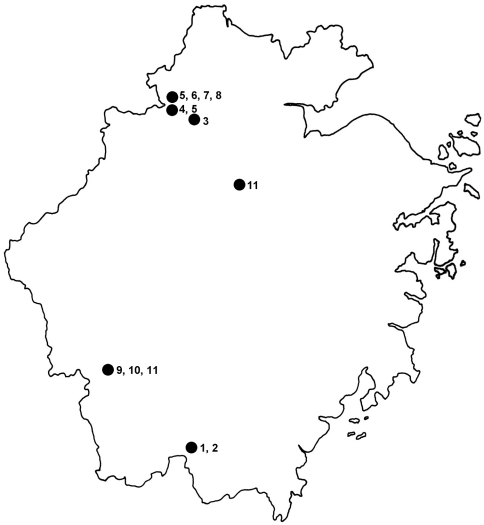
Distribution of the *Lathrobium* species in Zhejiang. **1** *Lathrobium imadatei* Watanabe & Luo **2** *Lathrobium tamurai* Watanabe & Luo **3** *Lathrobium cooteri* Watanabe **4** *Lathrobium rougemonti* Watanabe **5** *Lathrobium tianmushanense* Watanabe **6** *Lathrobium lingae* Peng, Li & Zhao **7** *Lathrobium longwangshanense* Peng, Li & Zhao **8** *Lathrobium uncum* Peng, Li & Zhao **9** *Lathrobium jiulongshanense* sp. n**.** **10** *Lathrobium sheni* sp. n. **11** *Lathrobium zhaotiexiongi* sp. n.

##### Key to the *Lathrobium* species of Zhejiang Province

**Table:** 

1	Length of body no more than 8.0 mm	2
–	Length of body larger than 8.5 mm	6
2	Male sternite VIII with symmetric emargination in posterior portion	3
–	Male sternite VIII with asymmetric emargination in posterior portion	5
3	Male sternite VII lacking short darkish setae; male sternite VIII (Fig. 3E) with two rows of long setae; BL 6.12–7.51	*Lathrobium sheni* sp. n.
–	Male sternite VII with short darkish setae; chaetotaxy of male sternite VIII different	4
4	Male sternite VIII with sparse modified setae in shallow impression; aedeagus with hook-shaped ventral process. BL 5.35–5.93	*Lathrobium uncum* Peng, Li & Zhao
–	Male sternite VIII with dense modified setae in deep impression; aedeagus with long and slender ventral process. BL 6.88	*Lathrobium lingae* Peng, Li & Zhao
5	Aedeagus with hook-shaped ventral process apically and broad dorsal sclerite. BL 5.90–6.30	*Lathrobium tamurai* Watanabe & Luo
–	Aedeagus with apically straight ventral process and narrow dorsal sclerite. BL 5.60–6.50	*Lathrobium rougemonti* Watanabe
6	Male sternite IV (Fig. 2D) with postero-median concavity. BL 8.90–10.08	*Lathrobium jiulongshanense* sp. n.
–	Male sternite IV without postero-median concavity	7
7	PL/PW ≥ 1.30; male sternite VI without sexual characters. BL 9.80–10.50	*Lathrobium cooteri* Watanabe
–	PL/PW ≤ 1.20; male sternite VI with sexual characters	8
8	Male sternite VI with U-shaped posterior impression, aedeagus with short and broad dorsal sclerite. BL 8.70–9.00	*Lathrobium imadatei* Watanabe & Luo
–	Male sternite VI with rounded postero-median concavity, aedeagus with dorsal sclerite of different shape	9
9	Male sternite VI with tuft of pubescence at concavity; aedeagus with long ventral process. BL 9.56	*Lathrobium longwangshanense* Peng, Li & Zhao
–	Male sternite VI lacking tuft of pubescence at concavity; aedeagus with short ventral process	10
10	Male sternite VII with distinctly asymmetric emargination in posterior portion; aedeagus (Fig. 4E, 4F) with short dorsal sclerite. Posterior margin of female tergite VIII (Fig. 4B) symmetric. BL 9.90–11.12	*Lathrobium zhaotiexiongi* sp. n.
–	Male sternite VII with weak and symmetric emargination in posterior portion; aedeagus with long dorsal sclerite. Posterior margin of female tergite VIII weakly asymmetric. BL 9.40	*Lathrobium tianmushanense* Watanabe

## Supplementary Material

XML Treatment for Lathrobium jiulongshanense

XML Treatment for Lathrobium sheni

XML Treatment for Lathrobium zhaotiexiongi
